# Freehand Pre-drilling Technique for Lateral Mass Screw Fixation Is More Efficient and Reliable Versus Sequential Drilling Technique: A Sawbone Analysis

**DOI:** 10.7759/cureus.33015

**Published:** 2022-12-27

**Authors:** Daren J McCalla, Takashi Hirase, Jacob C Hoffmann, Jeffrey J Ratusznik, Bradley Lambert, Rex Marco

**Affiliations:** 1 Spine Surgery, Houston Scoliosis and Spine Institute, Houston, USA; 2 Orthopedics and Sports Medicine, Houston Methodist Hospital, Houston, USA; 3 Orthopedic Surgery, Cleveland Clinic Akron General, Akron, USA

**Keywords:** spine surgery, sawbones, surgical technique, lateral mass screw, posterior cervical fixation

## Abstract

Background

Since posterior cervical fixation with lateral mass screws was introduced in 1979, multiple techniques have been described in the literature. However, no study to date has determined whether pre-drilling all lateral masses prior to screw insertion has a benefit over the traditional sequential drilling and screw insertion on the alignment of the screw-rod construct. This study sought to determine the efficacy and efficiency in achieving alignment with a novel pre-drilling technique compared to the traditional sequential drilling technique. The authors hypothesized that the novel pre-drilling technique could be applied more quickly and precisely than the traditional sequential drilling technique.

Methods

Eight cervical spine sawbones models were utilized to place 64 lateral mass screws by two surgeons. The pre-drilling technique was utilized to place 32 screws in four models, and the sequential drilling technique was utilized to place the 32 screws in the remaining four models. In the traditional sequential drilling technique, each lateral mass underwent screw tract preparation and insertion before proceeding to the subsequent vertebra. In the pre-drilling technique, all lateral masses were marked and drilled sequentially before screw placement. CT imaging with 3D reconstructions was generated for all models. Variability in screw placement and time taken to fully instrument the models were compared.

Results

The mean time to completion of the pre-drilling technique was 337 ± 22 seconds compared to 490 ± 22 seconds with the traditional technique (p<0.01). There was a significantly higher variability in the coronal plane within the traditional group between C5 and C6 compared to other adjacent vertebrae (p<0.05). There was no significant difference in the start point variability and the overall tightness of line fit between the techniques.

Conclusions

Our study suggests that a novel pre-drilling technique for lateral mass screw insertion may be more efficient and reliable than the traditional sequential drilling technique. In addition, this technique may reduce the need for rod contouring or additional implants to optimize the alignment of cervical instrumentation. However, further clinical studies are necessary to validate the potential clinical and radiologic benefits of this described technique.

## Introduction

Posterior cervical instrumentation constructs can safely realign the spinal column while achieving arthrodesis in various spinal disorders, including trauma, tumor, spondylosis, infection, inflammatory/rheumatologic disease, and other conditions [1]. Posterior cervical fixation with lateral mass screws has been used increasingly since first introduced by Roy-Camille in 1979 [2]. Lateral mass screw fixation provides multiple advantages over previously used systems, including amenability to multiplanar contouring, precision in placement of the lateral mass screws, and application of compression, distraction, and reduction forces within the system [3,4]. In addition to being efficacious, lateral mass screw fixation has infrequent complications, which include nerve root injury, vertebral artery injury, formation of pseudarthrosis, and screw pull-out [5,6]. Thus, the proper placement technique and a thorough understanding of the anatomy are crucial for the safe placement of an effective lateral mass screw.

Maximizing surgical efficiency while minimizing screw position variability are essential concepts for achieving optimal outcomes after posterior cervical instrumentation. Recent studies have found significant associations between longer operative time and postoperative length of stay, non-home discharge, and transfusion requirements after cervical spine surgery [7,8]. Furthermore, the importance of achieving appropriate rod contouring by minimizing screw position variability for optimizing biomechanical stability is a well-understood concept in spine surgery [9-11]. Consequently, utilizing a technique that reliably places safe lateral mass screws that both maximizes surgical efficiency and minimizes screw position variability is vital for favorable outcomes after posterior cervical instrumentation.

Although multiple screw placement techniques have been described in the literature, to our knowledge, no study to date has determined whether pre-drilling all lateral masses prior to screw insertion is beneficial over sequential drilling and screw insertion in regards to surgical efficiency and on the screw-rod construct alignment [2,12-14]. This study sought to determine the efficacy and efficiency in achieving alignment with a novel pre-drilling technique compared to the traditional sequential drilling technique. In addition, the authors hypothesized that the novel pre-drilling technique could be applied more quickly and precisely than the traditional sequential drilling technique.

## Materials and methods

The technique chosen for posterior cervical lateral mass fixation and screw trajectory was patterned after the technique that is used in the senior author’s practice (Rex Marco, fellowship-trained orthopedic spine surgeon). The aforementioned technique itself is an adaptation of the technique, which has been described and adopted by many spine surgeons [1,14]. An orthopedic spine fellow and an orthopedic resident were taught the senior author’s technique. After teaching and demonstration, the participants performed all screw tract preparation and instrumentation under the supervision of the senior author. All participants were right-hand dominant. 

Eight prone-positioned cervical spine sawbones models (Pacific Research Laboratories, Inc., Vashon, WA) were mounted onto a foam stabilizer and were categorized into four groups. The two techniques were each performed in two groups; one group was timed, and the other untimed. Technique 1 represents the traditional, sequential drilling and screw placement technique; Technique 2 represents the senior author’s preferred technique of pre-drilling all lateral mass screws pilot holes prior to screw insertion. 

Technique

First, the midsagittal and mid-axial intersection of the lateral mass was identified. Then, a marking was made using a marking pen at 1 mm medial and 1 mm caudal to the center of the quadrangular dorsal surface of the lateral mass. Next, a 2 mm pilot hole was created at this mark with a trajectory directed to the center of the lateral mass using a high-speed 3 mm x 3.8 mm burr. The 2.4 mm drill bit was then placed within the burr hole directed at the superolateral corner of the lateral mass. At this point, the techniques diverge: in Technique 1 (sequential drilling), the lateral mass of the first vertebra underwent pilot hole burring, followed by drilling, tapping of the screw tract, and then screw insertion, before moving to the subsequent vertebra. In Technique 2 (pre-drilling), all lateral masses were first marked before the pilot hole was created. The burr holes were then created sequentially from cranial to caudal. Then the screw tracts were drilled sequentially in the same fashion. Finally, the tract was tapped, and a screw was placed in each lateral mass, moving to the subsequent/caudad vertebra. Depuy-Synthes Synapse 3.5 mm x 14 mm polyaxial screws (Synthes, West Chester, PA) were inserted into the lateral masses of the C3, C4, C5, and C6 vertebrae of all models. All lateral mass screws were placed, and digital photographs of the models with a reference marker were taken in the coronal plane. The specimens were randomized by another blinded investigator prior to photographing and measuring. A straight line between the center of the screw holes at the C3 and C6 lines was made, and a similar line was extrapolated from a straight line between the C4 and C5 levels using the Image J software (NIH.com) program. The instrumented models were then imaged with CT, and 3D reconstructions were generated. Measurements for the screw start points were calculated as distance (in mm) from the pilot holes to the spinous process (representing the midline) and in relation to each other. The variability of screw start point placement in the transverse plane was compared between techniques by measuring the difference in the start point - spinous process distance across levels. The number of seconds taken to fully instrument C3-C6 bilaterally was also measured between techniques.

Statistical analysis

To compare shift/line of fit for the x-axis coordinate between screw points along adjacent vertebrae, a 3 (vertebrae segment; C3-C4, C4-C5, C5-C6) x 2 (technique) analysis of variance followed by a Tukey's post hoc test was performed. Additionally, a sum of squares test was performed to determine and compare the overall tightness of line fit from C3-C6 for both techniques. Finally, a student's T-test was used to compare procedure completion time between techniques. Type I error was set at α = 0.05 for all analyses. For all detected pairwise differences at p<0.05 and pairwise comparisons approaching significance at p<0.1, effect size (ES) is presented as Cohen's D statistic [Cohen's d = (Mean2 - Mean1) / SDpooled] whereby values are interpreted as follows: ES < 0.1, negligible; ES 0.1-0.3, small; ES 0.3-0.5, moderate; ES 0.5-0.7, larger; and ES > 0.7, very large.

## Results

**Figure 1:**
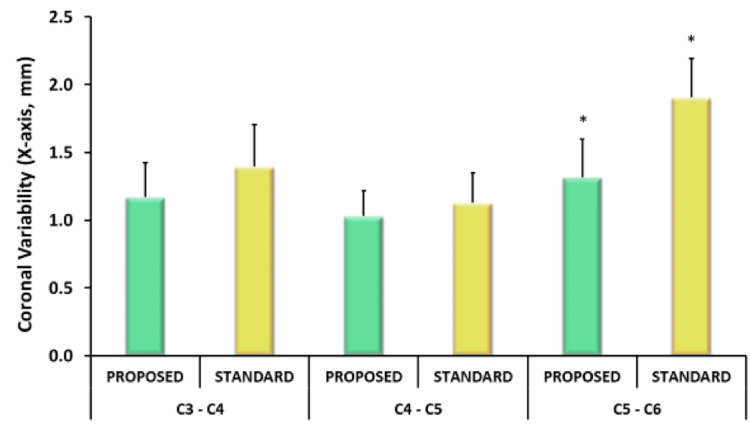
Data are presented as mean ± SEM for variability in the coronal plane between adjacent cervical spine vertebrae (*p<0.05). SEM: Standard error of mean.

Type III tests of fixed effects revealed an interaction of method and vertebral segment (p<0.05). Following post-hoc analysis, no significant difference was observed between techniques (p>0.05). However, the traditional sequential drilling technique demonstrated greater variability in the coronal plane between C3 and C4 (1.39+/-.31mm vs. 1.16+/-.25mm; p=0.11) and C5 and C6 (1.91+/-.29mm vs. 1.32 +/-.28mm; p=0.11) (Figure 1)

**Figure 2:**
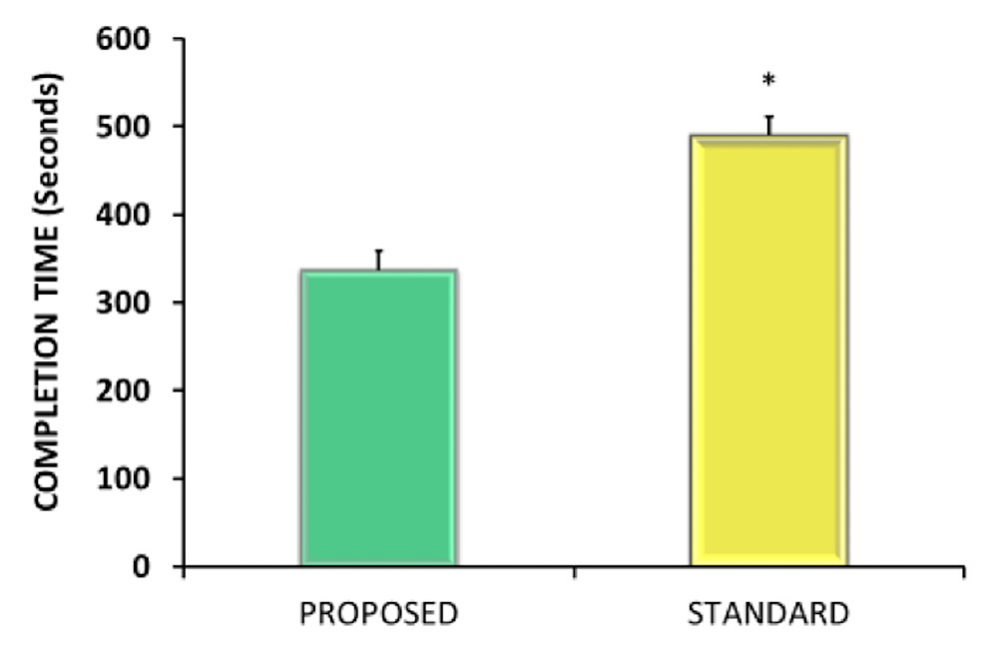
Data are presented as mean ± SEM for procedure duration (seconds) for the proposed standard techniques (*p<0.05). SEM: Standard error of mean.

When comparing the variability between vertebrae within a given technique between segments, no significant difference was observed within the novel pre-drilling technique group. In contrast, the traditional technique was found to have significantly higher variability between C5 and C6 compared to other adjacent vertebrae (p<0.05). Analysis of the sum of squares revealed no difference in the tightness of line fit between the techniques. The mean time to completion of the pre-drilling technique was 337 ± 22 seconds compared to 490 ± 22 seconds with the traditional technique (p<0.01) (Figure 2). 

## Discussion

In the present study, we explore a unique approach to optimizing the workflow of lateral mass screw placement while maintaining the effectiveness and safety of previously described methods. We believe this novel technique reduces the time needed to apply instrumentation and may improve screw alignment to facilitate rod placement. This novel approach represents a combination of the continuing advancements in the technology, knowledge, and skills applied to cervical spine surgery.

Roy-Camille's original description of posterior cervical stabilization with lateral mass screws acknowledged the relationship between the vertebral artery and the junction of the lamina and lateral mass (the "valley") [2]. The screw was placed in the center of the lateral mass, perpendicular to the sagittal plane of the lateral mass, and angled 10 degrees laterally to avoid the vertebral artery. Bicortical purchase of the screw was recommended, which could put the artery at risk with a malpositioned screw. This technique afforded robust fixation for cervical spine pathology but was not without shortcomings [1,2]. The exiting nerve root traveling anterior to the superior facet of the caudal vertebra could be injured by a bicortical Roy-Camille screw. Also, the inferior facet is at risk for violation with the screw trajectory, which is undesirable at the caudal end of the construct. Later techniques emphasized more superior angulation in the sagittal plane to mitigate these risks [1,12-15]. Superiorly angled bicortical screws may impact the exiting nerve root of the indicated vertebra (as opposed to the level below). However, the need for bicortical purchase is less necessary, given the ability to place longer screws via this path [1].

Recent research suggests that surgeons have difficulty precisely matching these expected angles with the naked eye [1,2]. While this imprecision has not been proven to have an adverse clinical effect, it is suggested that a more prudent approach would be to base screw trajectory on the given patient's anatomy, i.e., ipsilateral lamina [1,16]. Several recent studies support this anatomically based approach for its ease of application and safety [1,16,17]. Our trajectory technique is also simple and safe to apply, and we surmise that the same practical implications would apply in the setting of actual surgery.

We compared the absolute time needed to place bilateral lateral mass screws with the proposed technique versus a traditional method. We found that the proposed technique could be performed quicker due to minimizing unnecessary technical moves. To our knowledge, no previous study has compared lateral mass screw techniques in terms of operative time. It is well-known through the spine and general surgical literature that increased operative time is associated with increased complications such as infection, venous thromboembolism, pulmonary compromise, and hospital readmission [7,8,18,19]. One of the goals of the present study was to demonstrate that this technique can decrease the time and inefficiencies related to planning and placing screws, which may translate to decreased operative time in real practice. 

In the current study, we also sought to demonstrate that the proposed technique resulted in the more consistent placement of screws along a straight paramedian line. We believe that achieving this goal reduces the need for repeated contouring of rods or using additional implants, particularly in constructs that span the occipital-cervical or cervicothoracic junctions. It has been demonstrated in biomechanical and clinical studies that over-manipulation of rods can contribute to fatigue failure [9-11]. Furthermore, inserting additional implants may increase the operative time. The results in this study did not demonstrate that either technique significantly deviated from a best-fit line between the C3 and C6 lateral masses. However, the traditional technique lent itself to more inconsistent positioning of screws in the transverse plane. The reason for greater inconsistency in placing screws at the C5-C6 level is unclear but may be related to the surgeon's ability to adapt to the changing cervical lordosis. The implications of this finding would obviously vary with patient anatomy and pathology. However, it does suggest that optimizing the efficiency of motion in screw placement with the proposed technique may reduce the impact of those variables.

There are limitations in the current study that we acknowledge. While sawbones models are anatomically accurate simulations that have been used to augment surgical skills, they do not account for variations in soft tissue or structural anatomy nor dynamic factors that one encounters when performing cadaver or live patient operations [20,21]. In addition, we did not assess the accuracy of screw placement in the cephalad-caudad plane or trajectories as we did not feel this contributes significantly to the coronal alignment of the rod, which, as stated previously, can affect the need for contouring or additional implants.

## Conclusions

Our study suggests that a novel pre-drilling technique for lateral mass screw insertion may be more efficient and reliable than the traditional sequential drilling technique. In addition, this technique may reduce the need for rod contouring or additional implants to optimize the alignment of cervical instrumentation. However, further clinical studies are necessary to validate the potential clinical and radiologic benefits of this described technique.
